# Thermo- and pH-Responsible Gels for Efficient Protein Adsorption and Desorption

**DOI:** 10.3390/molecules29204858

**Published:** 2024-10-13

**Authors:** Izabela Poplewska, Beata Strachota, Adam Strachota, Grzegorz Poplewski, Dorota Antos

**Affiliations:** 1 Department of Chemical and Process Engineering, Rzeszów University of Technology, 35-959 Rzeszów, Poland; ichgp@prz.edu.pl (G.P.); dorota.antos@prz.edu.pl (D.A.); 2 Institute of Macromolecular Chemistry, Czech Academy of Sciences, 162 00 Prague, Czech Republic; beata@imc.cas.cz (B.S.); strachota@imc.cas.cz (A.S.)

**Keywords:** protein adsorption, protein release, hydrogels, pH sensitivity, temperature-sensitivity

## Abstract

Protein adsorption behavior was examined on poly(*N*-isopropylacrylamide-co-sodium methacrylate)-based hydrogels at different temperatures: 5, 20, and 37 °C, and pH: 4.5, 7, and 9.2. The hydrogels, whose covalent skeleton contains pendant anionic units due to the presence of the sodium methacrylate co-monomer, exhibited both thermo- and pH-sensitivity with different extents, which depended on the content of ionizable moieties and the cross-linker density. The hydrogel composition, temperature, and pH influenced the zeta potential of the hydrogels and their swelling properties. The proteins selected for the study, i.e., bovine serum albumin (BSA), ovalbumin (OVA), lysozyme (LYZ), and a monoclonal antibody (mAb2), differed in their aminoacidic composition and conformation, thus in isoelectric point, molecular weight, electrostatic charge, and hydrophobicity. Therefore, the response of their adsorption behavior to changes in the solution properties and the hydrogel composition was different. LYZ exhibited the strongest adsorption of all proteins with a maximum at pH 7 (189.5 $mg\;g_{gel}^{-1}$); adsorption of BSA and OVA reached maximum at pH 4.5 (24.4 and 23.5 $mg\;g_{gel}^{-1}$), whereas mAb2 was strongly adsorbed at 9.2 (21.7 $mg\;g_{gel}^{-1}$). This indicated the possibility of using the hydrogels for pH-mediated separation of proteins differing in charge under mild conditions in a water-rich environment of both the liquid solution and the adsorbed phase. The adsorption affinity of all proteins increased with temperature, which was attributed to the synergistic effects of attractive electrostatic and hydrophobic interactions. That effect was particularly marked for mAb2, for which the temperature change from 5 to 37 °C caused a twentyfold increase in adsorption. In all cases, the proteins could be released from the hydrogel surface by a reduction in temperature, an increase in pH, or a combination of both. This allows for the elimination of the use of salt solution as a desorbing agent, whose presence renders the recycling of buffering solutions difficult.

## 1. Introduction

The development of stimuli-responsive hydrogels, also called “smart” or “intelligent”, has attracted growing interest in many research areas. Smart hydrogels have also found a variety of applications in different fields, such as drug delivery systems, tissue engineering, sensors, diagnostics, imaging, and separation techniques [1,2,3,4,5,6,7,8,9,10,11,12,13,14,15,16]. They are characterized by biocompatibility and the ability for noncovalent interactions with biomolecules [12,17,18,19,20,21,22].

Smart hydrogels can respond to a variety of external stimuli, including temperature, pH, light, electric, and magnetic fields, incident light wavelength, or the ionic strength of the solution [8,9]. The response triggered by external stimuli has the form of a sharp transition with a drastic change in the structural properties of the hydrogel. Among smart hydrogels, thermo-responsive hydrogels are currently widely used. They can be stimulated by a slight shift in temperature, for example, from ambient to human body conditions, which induces precipitation in the case of a linear polymer or a distinct deswelling in the case of a crosslinked one (‘volume phase transition’) [1,23,24]. This creates potential for a variety of medical and biotechnology applications. Crosslinked poly(*N*-isopropylacrylamide) (PNIPAM) is a well-known thermosensitive hydrogel that exhibits marked deswelling above its lower critical solution temperature (LCST, around 32 °C) in aqueous media [23,24].

Due to their unique stimuli-responsive properties, PNIPAM-based hydrogels have been extensively examined for controlled protein adsorption and release [25,26,27,28]. The incorporation of pH-sensitive polymers into PNIPAM hydrogels lends them pH sensitivity as an additional property, which allows them to act as adsorbents for polyelectrolytes, counterions, proteins, and enzymes [29,30,31,32,33,34,35,36,37,38]. Protein adsorption and release on PNIPAM-based thermo- or pH-sensitive hydrogels have been extensively examined in a number of studies [25,26,27,28,39,40,41,42,43,44,45,46]. Above T = LCST, the thermo-responsive hydrogels become hydrophobic, favoring hydrophobic interactions as a driving force for protein adsorption [25,40]. The presence of charged moieties within the hydrogel structure causes the electrostatic interactions between the protein and the hydrogel to become stronger [47,48,49,50,51]. Charged protein molecules can undergo electrostatic interactions with charged surfaces of pH-sensitive hydrogels in synergy with the enhanced hydrophobicity of the hydrogels above LCST [41,42,43,44,45,46]. Adsorption of proteins on such dual-responsive hydrogels follows a complex mechanism based on a combination of hydrophobic and electrostatic interactions, hydrogen bonding, physical diffusion, and seizing effect [6,12,47,48,49,52,53,54,55,56,57]. The hydrogel composition can be altered to increase the contribution of the preferred interaction type to the adsorption mechanism. This allows the hydrogel to be tailored to the selective binding of target proteins. Protein adsorption and release on temperature- and pH-sensitive hydrogels containing poly(acrylic acid) (PAAc) PAAc-PNIPAM as a charged moiety has been investigated for BSA as a model compound [47,48,49,50,51]. The BSA molecule is negatively charged at a pH greater than its isoelectric point (pI), which is about 4.7. Therefore, electrostatic interactions between BSA and negatively charged PAAc-PNIPAM are active only at low pH. Because the adsorption mechanism is protein-specific, the adsorption trends reported in the above-referred studies could not be generalized. Hence, in this work, we investigated the adsorption behavior of four different proteins on temperature- and pH-responsive PNIPAM-co-PolySMA, in which sodium polymethacrylate moiety (SMA) was incorporated. The proteins selected for the study differed in pI and molecular weight and were lysozyme (LYZ), ovalbumin (OVA), monoclonal immunoglobulin (mAb2), and BSA as reference proteins. The purpose of this choice was to examine the possibility of pH-mediated selective adsorption of proteins with different charges. The effect of pH and temperature on the adsorption and release of proteins was determined for different compositions of PNIPAM-co-PolySMA hydrogels as a protein carrier.

## 2. Results and Discussion

### 2.1. Structure of the Tested Polymeric Adsorbent

**Figure 1:**
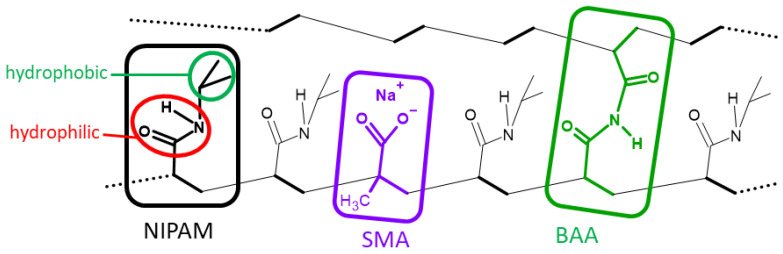
Structure of the tested polymer adsorbents (crosslinked PNIPAM-co-PolySMA), with highlighted monomeric units: NIPAM (the main monomer), responsible for LCST (T-induced switching between hydrophilic and hydrophobic state); SMA co-monomer responsible for swelling sensitivity to pH, which also means pH-dependent charge; BAA co-monomer incorporated as a crosslinker.

The structure of the polymer adsorbents (hydrogels) studied in the present work (crosslinked PNIPAM-co-PolySMA) is shown in Figure 1. The main monomer NIPAM is responsible for the temperature-sensitivity of the swelling of the hydrogels, that is, for the switching of T = LCST between the hydrophilic state (at T < LCST) and the hydrophobic state (at T > LCST) of the polymer. In this monomer, the CONH moieties are responsible for hydrophilic interactions, and the isopropyl groups on N are for the hydrophobic ones. As will be discussed in more detail below, the hydrophobic state was found to favor the adsorption of the tested proteins. The SMA co-monomer was responsible for the pH sensitivity of the swelling of the hydrogels. At high pH, in the deprotonated (ionic) highly hydrophilic state, it favors increased swelling. At low pH, in the protonated (non-ionic acid form), SMA is less hydrophilic, so the pH drop from high to low values causes the gel to shrink. An important aspect is that the mentioned ionization/protonation of SMA also leads to a change in the electrostatic charge of the polymer, which was found to be very important in the adsorption of the studied proteins. SMA (especially its anionic form) also influences the hydrophilicity of the whole polymer; it up-shifts the value of LCST [58].

The BAA crosslinker is responsible for the mechanical stability of the hydrogels studied, and it also limits the maximum swelling. In the case of low-crosslinked and thus highly swelling hydrogels, the ionic SMA units are markedly less concentrated (number of SMA groups per area unit) on the hydrogel surface in the expanded swollen state than in the shrunken one. On the other hand, in highly crosslinked hydrogels, changes in the surface concentration of SMA in response to T/pH stimuli are small because the possible changes in the swelling degree are small. At high crosslinker contents (e.g., at the maximum tested one: 20 mol% of BAA), the hydrogels were fairly rigid. The discussed BAA effects influenced the adsorption of proteins on the studied gels, as is discussed further below.

In order to confirm the chemical purity of the presently synthesized PNIPAM-co-PolySMA hydrogels, FT-IR spectra were measured, which are depicted in Figure S1 in Supplementary Materials. The spectra of the pure repeat units, PNIPAM and PolySMA, are overlaid in the same figure. The presented results of the measurements confirm the high purity of the prepared copolymers.

### 2.2. Selection of the Hydrogel Compositions and the Adsorption Conditions

The adsorption behavior of the proteins on the PNIPAM-co-PolySMA hydrogels and the hydrogel properties, such as swelling degree and zeta potential, were determined for different pH (4.5, 7, 9.2) and different temperatures (5, 20, 37 °C). The composition of the hydrogels was adjusted to alter their charge and hydrophobicity (Section 3.2). The increase in the content of the BAA co-monomer (5–20 mol%) led to a higher cross-linking density and thus to a reduced degree of swelling, while the increase in the content of the ionizable SMA co-monomer (2–10 mol%) caused higher ionic charges and hydrogel hydrophilicity. Mild pH conditions were selected for the present study, i.e., within the pH range of 4.5–9.2, which is typical for processing charged proteins by adsorption or chromatography. pH 4.5 was below the pI of all proteins examined; therefore, all of them were positively charged to a greater or lesser extent, depending on their pI and MW. Thus, they were prone to attractive electrostatic interactions with the PNIPAM-co-PolySMA hydrogels, which were negatively charged over the whole pH range examined (Section 2.4). At pH 7, only LYZ and mAb2 were positively charged, as their pI was above pH 7, and they could be attracted by ionized sites on the hydrogel surface. At pH 9.2, only LYZ was positively charged, while BSA, OVA, and mAb2 were negatively charged, and repulsive electrostatic interactions contributed to their binding mechanism. The selected temperature range, i.e., 5–37 °C, was also typical for protein processing. The temperatures of 5 °C and 20 °C were below LCST, while the temperature of 37 °C was in the vicinity of LCST for PNIPAM, which is typically around 32 °C. However, the LCST shifts to a higher temperature with increasing SMA content, to ca. 36 °C with 3 mol% SMA and up to ca. 40 °C with 10 mol% SMA, as documented in the literature, e.g., in [58].

### 2.3. Effect of the Composition, Temperature, and pH on the Swelling Degree of Hydrogels

**Figure 2:**
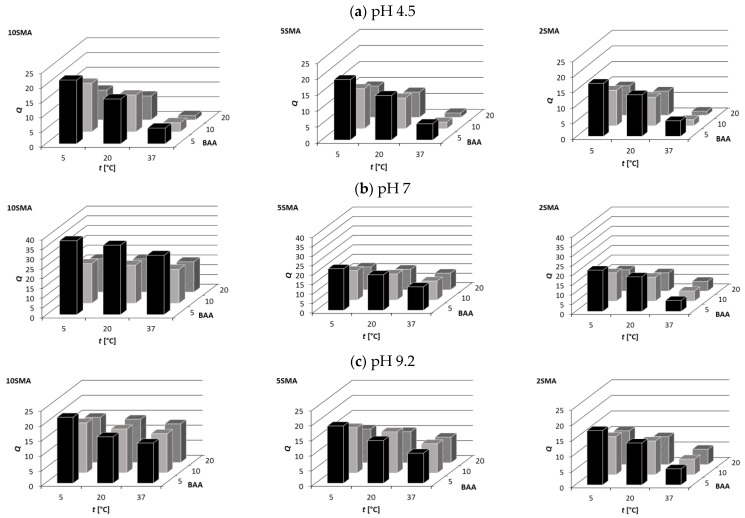
Temperature and composition dependence of *Q* for PNIPAM-co-PolySMA for different SMA and BAA content at pH 4.5 (**a**), 7 (**b**), and 9.2 (**c**).

The swelling behavior of the studied hydrogels plays an important role in their adsorption behavior (hydrophilicity, concentration of functional groups on the surface), as well as in their mechanical properties. The swelling degrees of the PNIPAM-co-PolySMA hydrogels measured at different pH and temperatures are visualized in Figure 2, while the exact data are presented in Table S1 in the Supplementary Materials.

As evident from Figure 2 and Table S1, the value of *Q* decreases with increasing temperature since the hydrogels contain the temperature-sensitive (LCST-type) PNIPAM polymer as the main component. The most cross-linked hydrogel that contains 20 mol% of BAA shows the lowest *Q* at 37 °C. For that hydrogel, the influence of the SMA content on *Q* was negligible as a result of a high network density. As expected, the hydrogel samples with the largest enrichment in the hydrophilic SMA moiety exhibited the highest *Q* values at each temperature. At pH = 4.5, the SMA moiety in PNIPAM-co-PolySMA was dissociated to ca. 50% (pKa of SMA is 4.8), which increased the hydrophilicity of the hydrogel and thus its swelling propensity.

Similar trends can be observed for pH 7, but for all hydrogel samples and all temperatures, the values of *Q* were higher than those for pH 4.5. This can be explained by a high degree of dissociation (ca. 100%) of the SMA moiety at pH 7, which enhanced the hydrophilicity of the hydrogel. The temperature dependence of *Q* at pH 7 was weaker compared to that observed for pH 4.5. This can also be attributed to the high degree of ionization of SMA, which exerted a dominating effect on the swelling properties. Only at the lowest SMA content, the temperature sensitivity of the hydrogel became visible, particularly at the lowest BAA content. In such ‘soft network’ hydrogels (5BAA-2SMA), the small amount of SMA still was able to generate a considerable pH response, without at the same time disabling the swelling response to T.

The trend in the *Q* values reported above for pH 4.5 and 7 was also preserved for pH 9.2 (Figure 2c), but despite the complete dissociation of the SMA moiety, *Q* took values lower than for pH 7 and similar to those measured for pH 4.5. In strongly alkaline media, the effects of condensation of counterions and charge screening with excess Na^+^ ions cause suppression of polyelectrolyte swelling [59,60]. Therefore, the degree of hydrogel swelling measured at pH 9.2 was lower than at pH 7 but higher than in more acidic solutions at pH 4.5. Similarly to pH 7, the swelling degree of the SMA-rich hydrogels (10SMA) showed at pH 9.2 very small temperature sensitivity.

Comparing all the data reported in Figure 2, one can observe that the pH effect on swelling properties is the most pronounced for the lowest BAA content combined with the highest SMA content (e.g., 5BAA-10SMA).

### 2.4. Zeta Potential

As will be discussed in detail later, the effect of the charge of colloidal protein particles, as well as the adsorber polymer, was one of the key mechanisms acting during the adsorption/desorption of the studied proteins on the PNIPAM-co-PolySMA hydrogels. Therefore, the charge of the hydrogels and the tested proteins under different experimental conditions was determined via the measurement of zeta potentials.

The zeta potential (***Z***) for the PNIPAM-co-PolySMA hydrogels was measured for different temperatures and pH of the solution, the same as those reported in Section 2.2. The results of the measurements are presented in Table 1.

As expected, *Z* of the hydrogels was negative, and its absolute value increased with increasing pH, which can be attributed to enhancing dissociation of carboxylic acid groups of the SMA moiety. For the same reason, the absolute values of *Z* increased with increasing content of the SMA co-monomer.

The influence of temperature on *Z* was negligible; the differences between the values of *Z* measured for the same hydrogel samples at different temperatures fell within the experimental errors. As reported in [61,62], the absolute value of *Z* increases for temperatures higher than LCST since a more collapsed surface promotes the adsorption of ions. But, as reported in [63], the effect of temperature on *Z* diminishes with increasing ionic strength of the solution. Hence, the presence of ions in the buffered solutions used in this study probably suppressed the temperature effect.

The hydrogels with the higher BAA content exhibited higher absolute values of *Z*. This effect was negligible at pH 4.5 and was slightly enhanced with increasing pH. The hydrogels with higher BAA content were characterized by lower swelling degrees (Figure 2, Table S1), causing ionized groups to be more concentrated on their surface compared to hydrogels with the same SMA content but with higher swelling due to lower crosslinking [64].

The zeta potential of the protein solutions was measured at the same temperature and pH 4.5, 7, and 9.2 at 20 °C. The results of the measurements are presented in Table 2.

### 2.5. Adsorption Properties of the Proteins on PNIPAM-co-PolySMA

**Figure 3:**
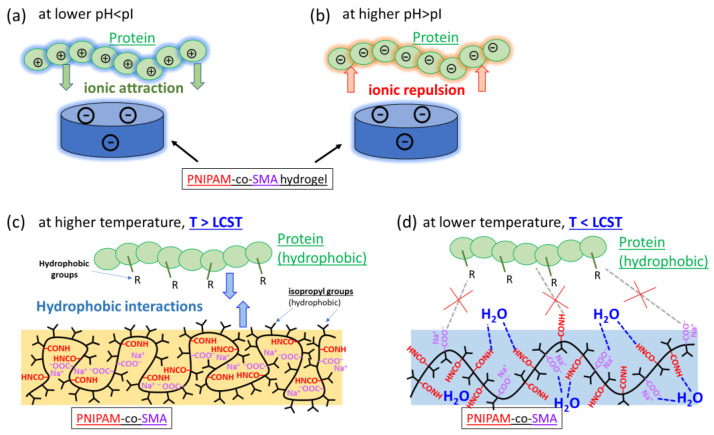
Cartoon representation of interactions between a protein and hydrogel (**a**) at a lower pH, (**b**) at a higher pH, (**c**) at a higher temperature (T > LCST), and (**d**) at a lower temperature.

The adsorption of the model proteins: BSA, OVA, LYZ, and mAb2, was measured on PNIPAM-co-PolySMA hydrogels of different compositions at different pH and temperatures of the solutions, as reported above. The analysis of the results indicates that in all the studied cases, two mechanisms simultaneously played a key role in the adsorption and desorption of the proteins on the PNIPAM-co-PolySMA hydrogels (see Figure 3): electrostatic interactions protein–gel (Figure 3a,b) and hydrophobic interactions protein–gel (Figure 3c,d). In the case of the PNIPAM-co-PolySMA hydrogels, the latter became hydrophobic above LCST (here 32 to 36 °C) while being hydrophilic below this temperature. The hydrogels became negatively charged at a pH close to, or higher than, the pKa of the SMA units (which is equal to ca. 4.8). The proteins, in turn, were positively charged at pH values smaller than their pI values. Combinations of attractive properties of gel and protein under the given experimental conditions led to adsorption, while those that were repulsive or indifferent favored desorption (see Figure 3).

#### 2.5.1. Adsorption Pattern of BSA and OVA

**Figure 4:**
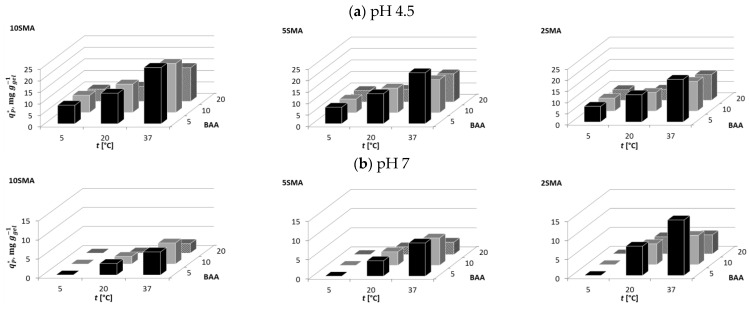

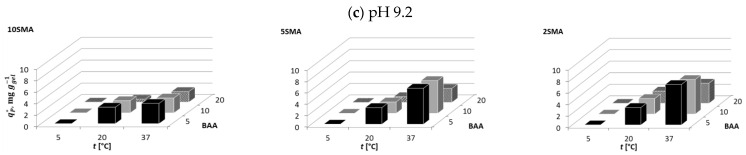
Illustration of the adsorption behavior of BSA on PNIPAM-co-PolySMA for different SMA and BAA content at pH 4.5 (**a**), 7 (**b**), and 9.2 (**c**).

At pH 4.5, the molecules of BSA were positively charged to a small extent (Table 2); therefore, they could be attracted by the PNIPAM-co-PolySMA hydrogels that were negatively charged (Table 2). As will be discussed below, the hydrophobic interactions between the hydrogels and the proteins additionally proved to be the second key factor in the adsorption tests. The dependencies of the adsorption affinity of BSA on temperature and the PNIPAM-co-PolySMA composition, i.e., on the content of SMA and BAA, are illustrated in Figure 4, while the exact data are presented in Table S2 in Supplementary Materials.

From Figure 4a, it can be observed that although BSA with pI = 4.7 was only slightly positively charged at pH 4.5, its adsorption on the hydrogels was possible. The adsorption became more prominent with the increasing SMA content, which indicated the occurrence of ionic interactions between the protein and the hydrogel surface. The adsorption strength decreased with increasing BAA content; an increase in the crosslinking density caused a higher surface concentration of the ionized SMA groups, which reduced the hydrophobic interaction of the protein with the hydrogel. However, an advantage of the increase in the crosslinker content was the increasing rigidity of the polymer, which improved its mechanical stability. The adsorption strength increased with increasing temperature (T > LCST), which activated hydrophobic interactions between the hydrophobic patches on the protein surface and the hydrophobic groups of the hydrogels.

At pH 7, BSA was slightly negatively charged (Table 2), therefore it was very weakly adsorbed on the negatively charged PNIPAM-co-PolySMA (Figure 4b). However, the ionic repulsive effect of the SMA content on the adsorption affinity was small (see the zeta potential in Table 1). Only for hydrogels with the lowest SMA content (2SMA), the adsorption was more significant but still much weaker than at pH 4.5. Adsorption became more prominent with decreasing BAA content (dilution of repulsive charge) and with increasing temperature due to the possibility of stronger hydrophobic interactions at T > LCST. At 5 °C, adsorption of BSA was negligible.

Analogous trends were observed for pH 9.2 like at pH 7: very weak adsorption of BSA, small effect of the SMA content, slight enhancement of adsorption with increasing temperature, and lack of adsorption at 5 °C (Figure 4c).

The results of the adsorption measurements for OVA are presented in the Supplementary Materials in Table S3 and Figure S2. It can be seen that the adsorption of OVA, whose pI = 4.6 is very similar to pI = 4.7 of BSA, followed very similar trends as in the case of BSA; adsorption was noticeable only at pH 4.5, with similar dependencies on temperature and polymer composition. For all conditions, the adsorption affinity of OVA was smaller compared with that of BSA. OVA has a smaller molecular size and therefore is less charged than BSA, which is reflected in its *Z* value (Table 2).

#### 2.5.2. Desorption of BSA

The effectiveness of BSA desorption was examined for hydrogels with the highest and lowest SMA content and with the lowest BAA content, i.e., 5BAA-10SMA and 5BAA-2SMA. The protein was adsorbed at 37 °C and pH 4.5, at which conditions its adsorption was the strongest, and, subsequently, it was desorbed at 5 °C or 37 °C, at pH equal to 4.5, or 7 or 9.2. Table 3 presents the protein recovery. It is evident that the release of BSA protein requires both an increase in pH and a decrease in temperature. The increase in pH weakened the electrostatic interaction (negative charge both in hydrogel and in protein), whereas the lowering of the temperature reduced the attracting hydrophobic interactions (at low T: hydrophilic gel + hydrophobic protein).

#### 2.5.3. Adsorption Pattern of LYZ

**Figure 5:**
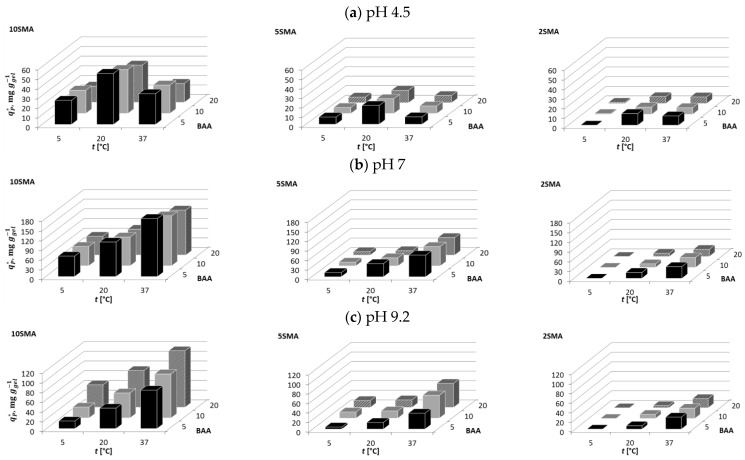
Illustration of the adsorption behavior of LYZ on PNIPAM-co-PolySMA for different SMA and BAA content at pH 4.5 (**a**), 7 (**b**), and 9.2 (**c**).

For all pH conditions tested (4.5, 7, 9.2), the molecules of LYZ were positively charged (Table 2), therefore they could be attracted by ionized (partly or fully deprotonated) SMA groups on the surface of the hydrogels surface (the hydrogels’ surface was always negative under the mentioned pH conditions). The adsorption pattern in terms of the adsorbed phase concentration is visualized in Figure 5, and the exact data are presented in Table S4 in the Supplementary Materials.

At pH 4.5, for each temperature and each hydrogel composition, the adsorption of LYZ increased with increasing SMA content, while it decreased with increasing BAA content. The adsorption affinity was the smallest at 5 °C and the highest at 20 °C. The unexpected reduction in adsorption affinity at 37 °C was found to be caused by structural changes in the protein upon adsorption. At the relatively low pH value of 4.5 and elevated temperature, the delicate (under this condition) LYZ structure denatured upon adsorption. This effect was proven by the SEC analysis of the protein solution after its contact with the hydrogel at pH 4.5; the peak of the processed protein significantly broadened in comparison with the peak of the native LYZ form, which indicated the appearance of new protein structures of various molecular weights corresponding to fragments of the denatured protein (see Supplementary Materials, Figure S3).

At pH 7, which was a mild condition for the binding of LYZ, its adsorption was the strongest. It improved with increasing temperature (hydrophobic interactions), with increasing content of SMA (ionic attraction), and with decreasing content of BAA (higher flexibility of polymer chains in the gel).

A similar trend was found for pH 9.2 as in the case of pH 7, although the adsorption was weaker than at pH 7 due to the reduction in the charge of the LYZ molecule, which has a pI value of 11.4. The only difference was the effect of the BAA content on the adsorption affinity; at pH 9.2 for 5SMA and 10SMA, the adsorption enhanced with increasing BAA content, which was particularly evident at higher temperatures. It is possible that the lower swelling degree of the highly crosslinked hydrogel led to a stronger hydrophobic interaction with the less diluted PNIPAM polymer, while the surface charge density on the gel was high. Compared to the situation at pH 7, it might be suggested that the hydrophobic effect played a more important role. The ionic strength at pH 9.2 could also have favored the clustering of charged groups in the warm hydrophobic hydrogel, which also led to the observed reduced swelling compared to pH 7 (Table S1). A case of similar behavior was also reported in the literature [12].

#### 2.5.4. Desorption of LYZ

Desorption of LYZ was examined for the hydrogels with the highest and lowest SMA content combined with the lowest BAA content, i.e., 5BAA-10SMA and 5BAA-2SMA. The protein was adsorbed at 37 °C, at pH 7 or pH 9.2, under which conditions the protein adsorption was strong, and it was subsequently desorbed at 5 °C or 37 °C, at pH values of 7 or 9.2. Table 4 presents the results of the recovery of the protein bound to the hydrogels.

It is evident that the release of LYZ previously adsorbed at pH 7 or pH 9.2 requires a reduction of temperature. For hydrogels with the lowest SMA content, on which hydrophobic interactions could strongly contribute to the adsorption mechanism, the lowering of the temperature to 5 °C was sufficient for complete recovery of the adsorbed LYZ. However, in the case of the hydrogels with the highest SMA content (10SMA), the protein recovery was incomplete. The increase in pH markedly improved the recovery, but still, a small amount of LYZ remained in the adsorbed phase. Complete desorption could only be achieved in the presence of salt ions (i.e., NaCl), which efficiently competed with the protein for the ionic adsorption sites.

#### 2.5.5. Adsorption of mAb2

**Figure 6:**
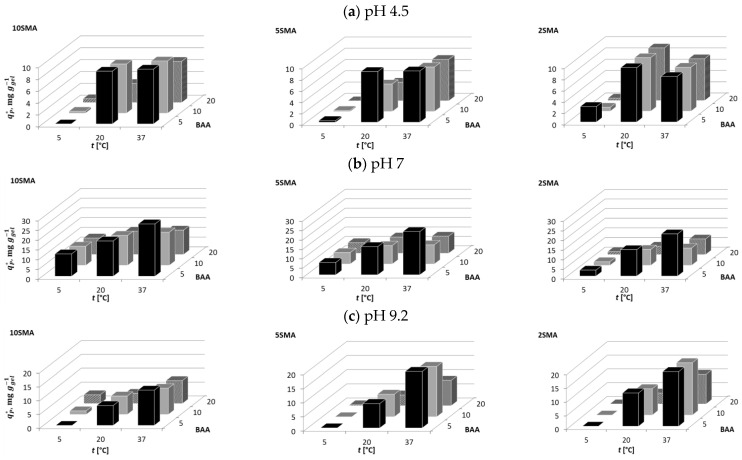
Illustration of the adsorption behavior of mAb2 on PNIPAM-co-PolySMA for different SMA and BAA content at pH 4.5 (**a**), 7 (**b**), and 9.2 (**c**).

The adsorption pattern of mAb2, which was positively charged at pH 4.5 and 7 and negatively charged at pH 9.2 (Table 2), is illustrated in Figure 6. The exact data are presented in Table S5 in Supplementary Materials.

Although the positive charge of mAb2 was the highest at pH 4.5 (see the zeta potential in Table 2 further above), its adsorption affinity was the smallest at this pH, compared to the one measured at pH 7 or at pH 9.2 (Figure 6a). The mAb2 adsorption was very weak or negligible at 5 °C, while it enhanced with increasing temperature to some extent. The strongest adsorption was observed at 20 °C for the hydrogel with the lowest SMA content. Interestingly, a further increase in temperature to 37 °C reduced the adsorption affinity. The reason for this unusual behavior was the same as that previously reported for LYZ adsorbed at pH 4.5; the protein structure destabilized at pH 4.5 in contact with the hydrogel, which was confirmed by the SEC analysis.

At pH 7, the adsorption of mAb2 increased with increasing SMA content and with increasing temperature, while it decreased with increasing BAA content, particularly at its highest value of 20 mol% of BAA (Figure 6b).

At pH 9.2, the temperature dependence of the adsorption strength of mAb2 was very strong; adsorption was negligible at 5 °C and increased significantly with increasing temperature (Figure 6c). Since pH 9.2 was above the pI value of the protein, the adsorption at this pH was driven by hydrophobic interactions, which had to dominate over the electrostatic repulsion; it was the strongest at the lowest SMA content and the weakest at the highest BAA content.

#### 2.5.6. Desorption of mAb2

The desorption efficiency of mAb2 was measured for the hydrogels 5BAA-10SMA and 5BAA-2SMA in a manner similar to that used for BSA and LYZ. The protein was initially adsorbed at 37 °C and pH 7 or pH 9.2, and subsequently desorbed at 5 °C or 37 °C, at pH 7 or 9.2. Table 5 presents the protein recovery in the desorption step.

mAb2 adsorbed at pH 7 could be recovered by a decrease in temperature combined with an increase in pH. For mAb2 adsorbed at pH 9.2, for which the desorption mechanism was mainly driven by hydrophobic interactions, the lowering of temperature to 5 °C was sufficient for the complete recovery of mAb2.

## 3. Materials and Methods

### 3.1. Materials

Bovine serum albumin (BSA), isoelectric point pI = 4.7, molecular weight MW = 66.4 kDa (purity ≥ 96%), lysozyme from chicken egg white (LYZ), pI = 11.4, MW = 14.4 kDa (purity ≥ 90%), and ovalbumin (albumin from chicken egg white) (OVA), pI = 4.6, MW = 44.3 kDa (purity ≥ 98%) were purchased from Sigma-Aldrich (Poznań, Poland). A monoclonal immunoglobulin (mAb2) with pI = 8.2, MW = 148 kDa (purity > 99%) was provided by Polpharma Biologics (Gdańsk, Poland). The stock solutions contained mAb2 with a concentration of 22 mg mL^−1^ in sodium acetate buffer (SA) 40 mM, pH 5.

*N*-isopropylacrylamide (NIPAM), sodium methacrylate (SMA), *N*,*N*′-methylenebisacrylamide (BAA), *N*,*N*,*N*′,*N*′-tetramethylethylenediamine (TEMED), and ammonium peroxodisulfate (APS) were purchased from Sigma-Aldrich and used as received. Acetic acid, sodium acetate, sodium chloride, sodium phosphate dibasic, and phosphoric acid were obtained from POCH.

### 3.2. Synthesis of the Hydrogels

PNIPAM-co-PolySMA hydrogels chemically cross-linked with BAA were obtained by free radical polymerization. NIPAM, BAA, and SMA were dissolved in distilled water. The homogeneous aqueous solution of the monomers was cooled to +15 °C and purged with argon. The redox co-initiator TEMED was subsequently added and stirred for approx. 1 min. Afterward, the initiator APS was admixed in the form of a 1% aqueous solution, which initiated the polymerization. The reaction mixture was immediately transferred to argon-filled tubes with an inner diameter of 1 cm and allowed to undergo radical polymerization at 25 °C for 24 h.

The amounts of components used to prepare the samples are given in Table 6. The concentration of double bond C=C in the reaction mixture was always 0.75 mol L^−1^. The concentration of SMA varied and was 2, 5, 10 mol% of the SMA double bonds, with respect to all polymerizable double bonds. The concentration of the BAA crosslinker was 5, 10, 20 mol% with respect to all polymerizable double bonds. The concentrations of the initiator (APS), the activator (TEMED), and the monomers with C=C were correlated by the following molar ratios: *r*(APS) = (APS)/(monomers with C=C) and *r*(TEMED/APS) = (TEMED)/(APS). These ratios were as follows: *r*(APS) = 0.0087 and *r*(TEMED/APS) = 3.2.

Sodium methacrylate (SMA) was chosen as the non-acidic form of the methacrylic acid (pKa 4.8) to avoid interference of the acidic co-monomer with the TEMED component (a diamine) of the co-initiator system APS/TEMED. The non-protonated electron pairs of TEMED play a key role in the low-temperature redox co-initiation mechanism (see [65]). Indeed, the authors observed that the presence of acids slows the polymerization of NIPAM by APS/TEMED. Depending on pH, the SMA co-monomer units present in the post-synthetized hydrogel either stay in the deprotonated state (above pH 4.8) or are protonated to methacrylic acid repeat units (below pH 4.8), thus switching between a very highly hydrophilic ionic state and a non-ionic (and somewhat less) hydrophilic protonated state and making the hydrogel pH-responsive.

The synthesized gels belong to a family that is characterized by very high gel fractions, close to 100%. SMA-free bulk PNIPAM samples crosslinked just by 3 mol% of BAA were found to display a gel fraction of 100% in [64], and even physically crosslinked (BAA-free) P(NIPAM-co-SMA)/nanoclay hydrogels displayed gel fractions between 97 and 100%, depending on clay and SMA content [66]. In the latter case, the SMA co-monomer negatively influenced the self-assembly with clay. The presently studied gels do not display such anti-self-assembly effects, and they contain a larger amount of crosslinker than the products in [64]. The quantitative nature of the crosslinking co-polymerization of NIPAM, BAA, and SMA also means that the molar fractions of the co-monomers used in the synthesis mixtures (as listed in Table 6) are automatically the molar fractions in the final crosslinked polymer.

### 3.3. Fourier-Transform Infrared Spectroscopy (FTIR)

For chemical characterization of the synthesized PNIPAM-co-PolySMA, the FT-IR spectra were measured using the Thermo Scientific Nicolet 8700 spectrometer and KBr pellets. All samples were analyzed at room temperature in the wavenumber range of 4000 to 500 cm^−1^.

### 3.4. Determination of the Swelling Degree

The phase transition of PNIPAM-co-PolySMA was studied as a function of the polymer composition, pH, and temperature of the solution. Samples of the polymers with different contents of SMA and BAA were incubated at different pH, i.e., 4.5, 7.0, or 9.2, and at different temperatures, i.e., 5, 20, and 37 °C. To determine the swelling degree (*Q*) at 20 °C, the samples were conditioned at 20 °C, then filtrated, weighed, and dried to constant weight at the same temperature. To determine *Q* at 5 °C and 37 °C, the samples, which were previously conditioned and weighed at 20 °C, were covered with a proper buffer solution and incubated for 24 h at temperature 5 °C or 37 °C. The samples were then filtered and weighed at an adequate temperature. For each temperature, *Q* was determined as a mass ratio of the swollen and dry polymer, as follows:

(1) $$Q^{20{}^{\circ}C}=\frac{m_{w}^{20{}^{\circ}C}}{m_{d}}$$

(2) $$Q^{5{}^{\circ}C}=\frac{m_{w}^{5{}^{\circ}C}}{m_{d}}=\frac{m_{w}^{5{}^{\circ}C}\;Q^{20{}^{\circ}C}}{m_{w}^{20{}^{\circ}C}}$$

(3) $$Q^{37{}^{\circ}C}=\frac{m_{w}^{37{}^{\circ}C}}{m_{d}}=\frac{m_{w}^{37{}^{\circ}C}\;Q^{20{}^{\circ}C}}{m_{w}^{20{}^{\circ}C}}$$

where $m_{w}$ is the mass of the swollen gel (g) at a specified temperature, and $m_{d}$ is the mass of the dry gel (g).

### 3.5. Determination of the Zeta Potential

Before measuring the zeta potential, the hydrogels were dried to constant mass, and then ground in the SPEX Sample Prep 8000M ball mill at 1425 rpm using a steel grinding ball with a diameter of 10 mm. The size of the swollen particles obtained after conditioning at different pH 4.5, 7, or 9.2 and temperatures of 20 °C was analyzed by light microscopy (Bresser TRM-301, Germany). The hydrogel particles were approximately spherical with diameters below 100 μm, which remained within the measurement range of the device (3.8 nm–100 μm).

To measure the zeta potential, 1.6 mg samples of dried gels were covered with 2 mL of buffered solutions of pH 4.5, 7, or 9.2 and conditioned at 20 °C or 37 °C for 1 h. Next, samples of 0.8 mg mL^−1^ of the obtained suspensions were mixed with samples of the protein solutions with a concentration of 1 mg mL^−1^ and equilibrated at the specified temperatures for 5 min and subjected to the zeta potential measurements. The measurements were performed using the Zetasizer Nano ZS instrument (Malvern Panalytical, Malvern, UK). The zeta potential values were calculated from the measured potential values according to Smoluchowski’s equation. The measurements were repeated for blank samples free of hydrogel or free of the protein, i.e., for samples of buffers: 100 mM sodium acetate buffer (SA) with pH 4.5, or 50 mM disodium phosphate buffer (PB) with pH 7 or 9.2. The values of the zeta potential measured for the blank sample were subtracted from the values of the zeta potential obtained for the hydrogels or the protein solutions. Each measurement was performed in triplicate, and the average value was used for the calculations.

### 3.6. Measurement of Protein Adsorption

The PNIPAM-co-PolySMA hydrogels obtained after synthesis were cut into small pieces and subjected to conditioning in buffered solutions of proper pH, i.e., in SA pH 4.5, PB pH 7 or 9.2, for 24 h at room temperature (20 °C). The swollen particles were passed through a sieve with a mesh diameter of 0.2 mm to obtain particles of similar shape and size. The size of the samples of the swollen particles was analyzed by light microscopy Bresser TRM-301 (Bresser, Rhede, Germany).

The stock solutions of the proteins BSA, LYZ, and OVA were prepared in the same buffers (SA, PB). Before use, the mAb2 stock solutions were subjected to exchange for adequate buffer using Amicon^®^ Ultra Centrifugal Filter Units, with a volume of 15 mL and a molecular weight cut-off of 10 kDa (Merck Millipore, Burlington, MA, USA). To determine the mAb2 concentration, the Infinite 200 PRO multimode microplate reader (Tecan Group Ltd., Männedorf, Switzerland) was used.

In the next step, 1 mL of the protein stock solution with the concentration of 1 mg mL^−1^ was mixed with 0.1 g of the swollen gel and agitated for 2 h at 150 rpm at 5, 20, or 37 °C. Next, a sample of the supernatant was acquired using a syringe filter PES 0.2 μm (Alchem, Toruń, Poland) and subjected to the concentration analysis. For this purpose, the HPLC instrument Äkta purifier (GE Healthcare Life Sciences, Uppsala, Sweden) with the UV (at 280 nm) and conductometric detectors and a data station were used. The samples were injected directly into the detecting system using the Rheodyne sampling valve with a 0.1 mL sample loop.

The equilibrium concentration of the proteins was calculated using the detector calibration curves determined for each protein standard. The amount of the protein adsorbed on the gel was calculated from the following material balance equations:

(4) $$q_{P}^{*}=\frac{C_{P}^{0}V_{0}-C_{P}V}{m_{w}}Q^{20{}^{\circ}C}$$

where $C_{P}^{0}$ and *C_P_* is the initial and equilibrium concentration of the protein in the liquid phase (mg mL^−1^), respectively; $q_{P}^{*}$ is the equilibrium adsorbed phase concentration (mg per g of the mass of dry gel; $mg\;g_{gel}^{-1}$), *m_w_* is the mass of the swollen gel at 20 °C (g); *V*_0_ and *V* is the initial and equilibrium volume of the protein solution (mL), respectively. The value of *V* was calculated from the swelling degree, accounting for the amount of water released or absorbed as a result of the temperature change. All measurements were made in triplicate.

### 3.7. Measurement of Protein Desorption

The samples of PNIPAM-co-PolySMA with the adsorbed protein were filtered, washed with the binding buffer used for the adsorption step, and then covered with 1 mL of the appropriate buffer (SA, PB), conditioned at 37 °C or 5 °C for 2 h while stirring with a magnetic stirrer (150 rpm).

### 3.8. SEC Analysis

The SEC-HPLC analysis was performed using the DIONEX UltiMate 3000 system (Thermo Fisher Scientific, Germering, Germany) with a UV detector. The analytical column TSKgel G3000SWxL (TOSOH BIOSCIENCE, Griesheim, Germany) with a bed particle size of 5 μm, I.D. = 7.8 mm, length 30 cm, particle size 1.7 μm was used. The mobile phase was a 100 mM sodium chloride solution in 50 mM phosphate buffer pH 7.

## 4. Conclusions

Adsorption of bovine serum albumin (BSA), ovalbumin (OVA), lysozyme (LYZ), and monoclonal antibody (mAb2) was measured on thermo- and pH-responsive hydrogels based on cross-linked poly(*N*-isopropylacrylamide-co-sodium methacrylate). The hydrogels contained the ionizable moieties of sodium methacrylate SMA and the crosslinker in different molar fractions, which influenced the hydrogel properties, including the zeta potential and the equilibrium swelling degree. Changes in pH and temperature, which strongly affected the properties of the prepared hydrogels, caused markedly different responses to the absorption behavior of the aforementioned proteins. BSA and OVA, which possessed the lowest isoelectric points, could be efficiently adsorbed only at pH 4.5 (maximum 24.4 and 23.5 $mg\;g_{gel}^{-1}$). The adsorption of LYZ, which had the highest isoelectric point, was the strongest of all proteins, but at pH 4.5, detrimental changes in protein structure occurred, particularly at elevated temperatures. Therefore, its adsorption was the most efficient at pH 7 (maximum 189.5 $mg\;g_{gel}^{-1}$). Adsorption of LYZ was improved by increasing the content of SMA, which indicates a strong contribution of attractive electrostatic interactions to its adsorption. mAb2 exhibited a similar problem with structural changes at pH 4.5 as LYZ did; therefore, the adsorption of mAb2 was highest at pH 7 (maximum 26.8 $mg\;g_{gel}^{-1}$) in the gel with the highest SMA content, where electrostatic interactions dominated the adsorption mechanism. The strong adsorption of mAb2 was also achieved at pH 9.2 with the lowest SMA content (maximum 21.7 $mg\;g_{gel}^{-1}$), where hydrophobic interactions were dominant. For all proteins, the adsorption improved with increasing temperature, which indicated the importance of the hydrophobic mechanism in all cases. Therefore, in general, the adsorption of all studied proteins can be viewed to be based on a synergy between hydrophobic and electrostatic interactions. All studied proteins could be released from the hydrogel by a decrease in temperature down to 5 °C, by raising the pH, or by using both stimuli combined.

The considerable differences in the adsorption properties of the studied proteins indicated a possibility of their separation by altering the hydrogel composition, pH, and temperature of the solution. The possibility of adjustment of two independent process variables, i.e., pH and temperature, allows for improvement in the effectiveness of both separation and desorption of proteins compared with standard ion exchange-based separation techniques. The elimination of salt solutions, which are typically used for protein release in the desorption step, is a major benefit resulting from the use of thermo- and pH-responsive hydrogels. The presence of salt in protein solutions is an issue in protein processing, as it has to be removed by an additional desalting operation and causes issues in waste management.

## Figures and Tables

**Table 1:** Zeta potential of the PNIPAM-co-PolySMA hydrogels.

Zeta Potential, mV ±2 mV						
	pH 4.5		pH 7		pH 9.2	
	20 °C	37 °C	20 °C	37 °C	20 °C	37 °C
20BAA-10SMA	−5.5	−5.6	−17.2	−13.6	−18.8	−16.2
5BAA-10SMA	−5.9	−5.7	−11.3	−10.0	−13.6	−12.4
20BAA-5SMA	−2.4	−2.3	−7.2	−5.7	−8.8	−9.3
5BAA-5SMA	−2.1	−3.1	−2.6	−4.0	−6.6	−6.2
20BAA-2SMA	−2.2	−1.1	−2.8	−4.5	−5.6	−5.5
5BAA-2SMA	−1.4	−1.4	−2.4	−3.1	−2.5	−4.4

**Table 2:** Zeta potential of the proteins at 20 °C.

Zeta Potential, mV ±2 mV			
	pH 4.5	pH 7	pH 9.2
BSA	+7.3	−0.7	−6.8
OVA	+6.2	−0.6	−5.2
LYZ	+15.6	+4.9	+4.6
mAb2	+14.1	+5.3	−2.2

**Table 3:** Recovery of BSA adsorbed at pH 4.5 and 37 °C (expressed as the percentage of the protein mass released to the mass of the protein previously bound in the adsorption step).

Hydrogel	pH 4.5 5 °C	pH 7 37 °C	pH 7 5 °C	pH 9.2 5 °C
5BAA-10SMA	65.2	74.8	96.1	100
5BAA-2SMA	62.9	23.4	100	100

**Table 4:** Recovery of LYZ adsorbed at 37 °C, and pH 7 or 9.2.

Hydrogel	After Adsorption at pH 7			After Adsorption at pH 9.2
	pH 7 5 °C	pH 9.2 37 °C	pH 9.2 5 °C	pH 9.2 5 °C
5BAA-10SMA	64.8	55.5	89.2	79.3
5BAA-2SMA	100	28.2	100	100

**Table 5:** Recovery of mAb2 adsorbed at 37 °C, at pH 7 or 9.2.

Hydrogel	After Adsorption at pH 7			After Adsorption at pH 9.2
	pH 7 5 °C	pH 9.2 37 °C	pH 9.2 5 °C	pH 9.2 5 °C
5BAA-10SMA	55.4	50.1	100	97.6
5BAA-2SMA	84.6	~0	100	100

**Table 6:** Compositions of the prepared PNIPAM-co-PolySMA.

		2 mol% SMA		5 mol% SMA		10 mol% SMA	
		g	mmol	g	mmol	g	mmol
5 mol% BAA	H_2_O	14.51	805.9	15.00	832.9	15.89	882.1
	NIPAM	1.500	13.26	1.500	13.26	1.500	13.26
	BAA	0.055	0.356	0.057	0.368	0.060	0.390
	SMA	0.031	0.285	0.080	0.736	0.168	1.559
	TEMED	0.046	0.399	0.048	0.412	0.051	0.437
	1%APS	2.830	0.124	2.924	0.128	3.096	0.136
10 mol% BAA	H_2_O	15.37	853.2	15.91	883.4	16.91	938.9
	NIPAM	1.500	13.26	1.500	13.26	1.500	13.26
	BAA	0.116	0.753	0.120	0.780	0.128	0.828
	SMA	0.033	0.301	0.084	0.780	0.179	1.657
	TEMED	0.049	0.422	0.051	0.437	0.054	0.464
	1%APS	2.990	0.131	3.096	0.136	3.290	0.144
20 mol% BAA	H_2_O	17.40	966.0	18.10	1005	19.39	1077
	NIPAM	1.500	13.26	1.500	13.26	1.500	13.26
	BAA	0.262	1.699	0.273	1.767	0.292	1.894
	SMA	0.037	0.340	0.095	0.884	0.205	1.894
	TEMED	0.055	0.476	0.057	0.495	0.062	0.530
	1%APS	3.374	0.148	3.509	0.154	3.759	0.165

## Data Availability

Data are contained within the article and Supplementary Material.

## Supplementary Materials

The following supporting information can be downloaded at: https://www.mdpi.com/article/10.3390/molecules29204858/s1, Figure S1: FT-IR absorbance spectrum of the PNIPAM-co-SMA hydrogel containing 10 mol% SMA and crosslinked with 20 mol% BAA, PolySMA and PNIPAM; Figure S2: Illustration of the adsorption behavior of OVA on PNIPAM-co-SMA for different SMA and BAA content at different pH; Figure S3: SEC analysis of LYZ solution; Table S1: Values of the swelling degrees, Q, of PNIPAM-co-SMA with different compositions at different pH and temperatures; Table S2: Comparison of adsorption of BSA at different pH and temperatures; Table S3: Comparison of adsorption of OVA at different pH and temperatures; Table S4: Comparison of adsorption of LYZ at different pH and temperatures; Table S5: Comparison of adsorption of mAb2 at different pH and temperatures
