# Supplementary material for: Thermo- and pH-Responsible Gels for Efficient Protein Adsorption and Desorption
Source: Molecules. 2024 Oct 13;29(20):4858. doi: 10.3390/molecules29204858 (PMC11510233; doi:10.3390/molecules29204858)
Supplement: Supplementary file 1 [file molecules-29-04858-s001.zip › molecules-3217157-supplementary.pdf]

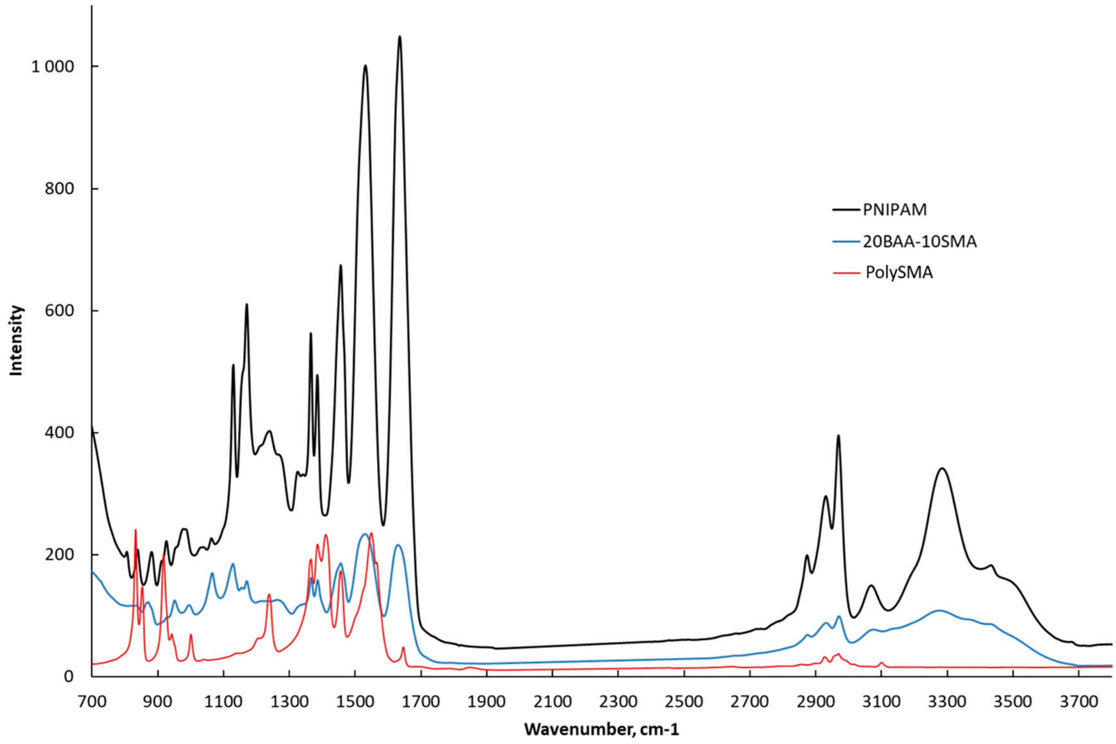

Figure S1. FT-IR absorbance spectrum of the PNIPAM-co-SMA hydrogel containing 10 mol% SMA and crosslinked with 20 mol% BAA (blue line) overlaid with the spectrum of neat PNIPAM (black line) and of neat PolySMA (red line); prominent IR peaks of neat PNIPAM, as well as of the copolymeric gel in which PNIPAm dominates, are: the N-H stretching bands of amides (3450, 3287 cm^{-1}), the characteristic amide bands in the fingerprint region, namely band I: amide C=O stretching (1634 cm^{-1}) and band II: combined C-N stretching and N-H bending of amides (1530 cm^{-1}), additionally the C-H stretching (3073, 2969, 2931, and 2871 cm^{-1}) and the C-H bending bands (1456, 1385, 1363 cm^{-1}) of the of $-CH_3$, $-CH_2-$, and $–C(CH_3)_2$ groups, and also the C-N stretching band (1169 cm^{-1}). It can be seen that all the prominent peaks of PolySMA overlap with prominent peaks of PNIPAM. It can be further observed that no additional impurity signals appear in the copolymer spectrum.

Table S1. Values of the swelling degrees, *Q*, of PNIPAM-co-SMA with different compositions at different pH and temperatures.

Q									
	**pH 4.5**			**pH 7**			**pH 9.2**		
	**5°C**	**20°C**	**37°C**	**5°C**	**20°C**	**37°C**	**5°C**	**20°C**	**37°C**
20BAA-10SMA	10.1	8.1	1.5	17.1	17.0	15.5	15.1	14.4	12.9
10BAA-10SMA	16.5	12.5	3.1	20.4	19.6	17.4	16.7	14.6	13.1
5BAA-10SMA	21.6	15.2	5.3	37.9	35.6	30.4	22.0	15.5	13.4
20BAA-5SMA	9.8	7.9	1.4	12.1	10.9	8.8	11.2	10.3	8.5
10BAA-5SMA	12.5	9.6	2.1	15.6	13.8	10.1	15.1	13.7	9.7
5BAA-5SMA	18.9	13.9	5.1	22.0	18.8	12.4	19.0	14.2	10.0
20BAA-2SMA	9.5	7.8	1.3	11.0	9.5	5.1	10.9	9.0	5.0
10BAA-2SMA	11.5	9.2	2.0	14.8	12.5	5.3	12.3	10.9	5.1
5BAA-2SMA	17.1	13.4	5.1	21.5	18.0	5.7	17.5	13.5	5.2

Table S2. Comparison of adsorption of BSA at different pH and temperatures.

q_P^*, mg g_{gel}^{-1}									
	pH 4.5			pH 7			pH 9.2		
	5°C	20°C	37°C	5°C	20°C	37°C	5°C	20°C	37°C
20BAA-10SMA	5.3	6.5	14.6	~0	0.3	2.5	~0	0.5	1.8
10BAA-10SMA	7.5	12.3	21.3	~0	2.0	5.5	~0	2.1	2.6
5BAA-10SMA	8.0	13.2	24.4	~0	3.0	6.0	~0	2.9	3.5
20BAA-5SMA	5.1	5.5	12.2	~0	2.1	3.2	~0	1.0	2.5
10BAA-5SMA	6.0	10.8	14.4	~0	3.5	7.1	~0	2.0	5.8
5BAA-5SMA	7.1	13.1	22.2	~0	4.0	8.6	~0	2.9	6.3
20BAA-2SMA	4.8	5.0	11.5	~0	4.5	5.0	~0	2.1	3.5
10BAA-2SMA	5.8	8.4	13.3	~0	5.5	7.7	~0	2.7	6.0
5BAA-2SMA	7.0	12.2	19.1	~0	7.7	14.6	~0	3.0	7.0

Table S3. Comparison of adsorption of OVA at different pH and temperatures.

q_P^* mg g_{gel}^{-1}									
	pH 4.5			pH 7			pH 9.2		
	5°C	20°C	37°C	5°C	20°C	37°C	5°C	20°C	37°C
20BAA-10SMA	4.8	5.9	13.2	~0	~0	1.8	~0	~0	1.4
10BAA-10SMA	6.8	11.5	20.6	~0	1.4	4.9	~0	1.5	2.3
5BAA-10SMA	7.5	12.8	23.5	~0	2.3	5.5	~0	2.1	3.0
20BAA-5SMA	4.2	4.9	11.7	~0	1.8	2.6	~0	0.5	2.1
10BAA-5SMA	5.5	10.1	13.6	~0	2.7	5.8	~0	1.7	4.6
5BAA-5SMA	6.5	11.9	20.1	~0	3.2	7.7	~0	2.2	5.5
20BAA-2SMA	3.9	4.4	10.8	~0	3.8	4.3	~0	1.6	2.9
10BAA-2SMA	5.3	7.6	12.8	~0	4.9	7.1	~0	2.0	5.5
5BAA-2SMA	6.0	10.8	16.0	~0	6.8	12.1	~0	2.5	6.4

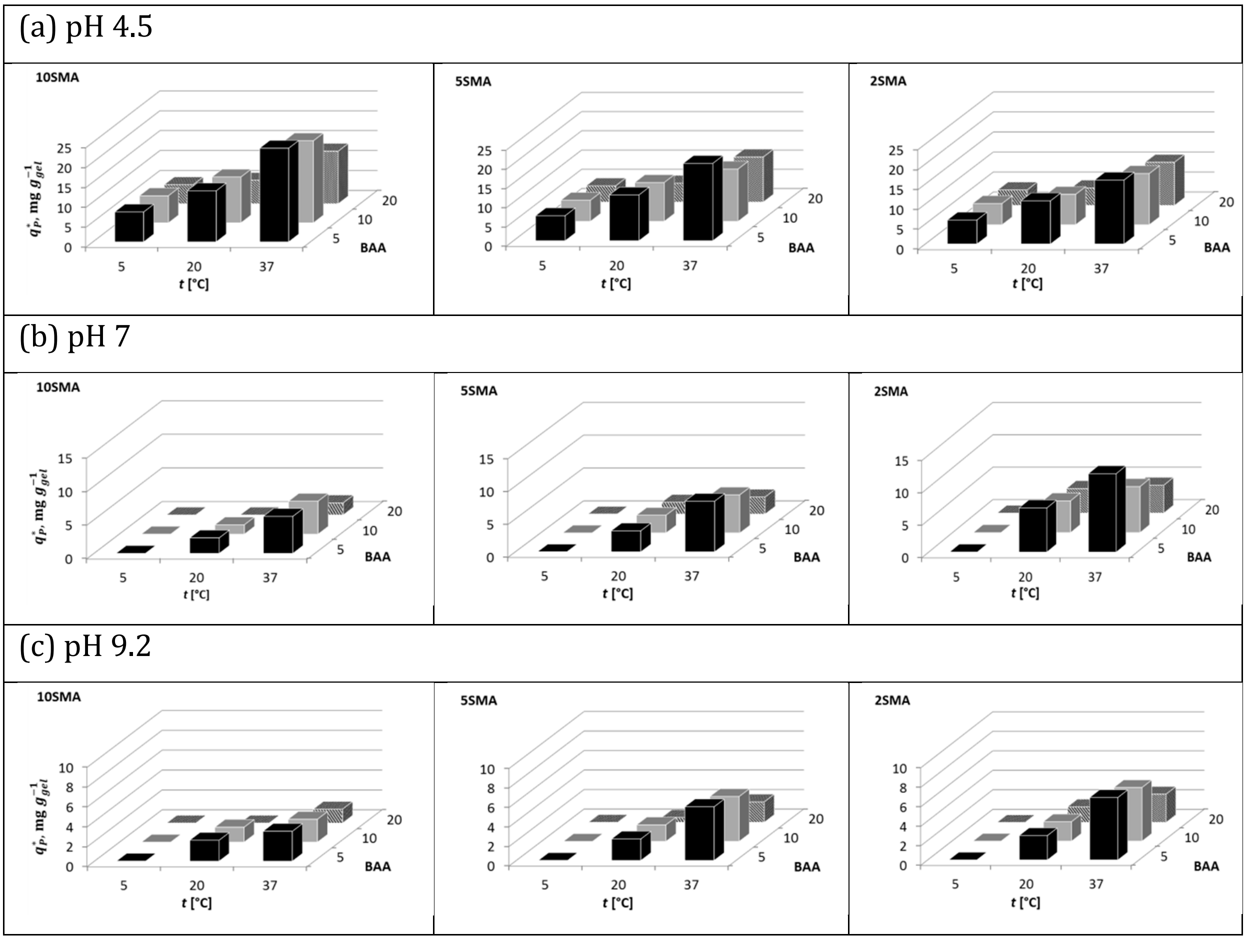

Figure S2. Illustration of the adsorption behavior of OVA on PNIPAM-co-SMA for different SMA and BAA content at pH 4.5 (a), 7 (b), and 9.2 (c).

Table S4. Comparison of adsorption of LYZ at different pH and temperatures.

	q_P^*, mg g_{gel}^{-1}								
	pH 4.5			pH 7			pH 9.2		
	5°C	20°C	37°C	5°C	20°C	37°C	5°C	20°C	37°C
20BAA-10SMA	16.8	38.6	19.6	58.3	80.0	139.5	46.4	75.6	116.3
10BAA-10SMA	24.1	45.6	29.4	60.0	89.3	155.0	22.4	51.7	90.7
5BAA-10SMA	25.0	53.1	31.9	63.3	107.0	189.5	15.6	42.2	79.4
20BAA-5SMA	6.0	13.1	7.6	11.0	13.8	55.2	14.4	15.8	50.4
10BAA-5SMA	6.3	15.5	7.9	12.0	25.7	61.2	13.0	15.3	48.0
5BAA-5SMA	7.6	19.7	7.8	13.1	41.3	68.0	4.1	13.8	33.3
20BAA-2SMA	1.1	7.0	6.8	~0	9.7	21.7	~0	4.6	19.2
10BAA-2SMA	0.8	7.6	6.9	~0	10.9	30.7	~0	8.5	21.4
5BAA-2SMA	~0	12.0	9.6	~0	18.2	36.0	~0	7.1	25.0

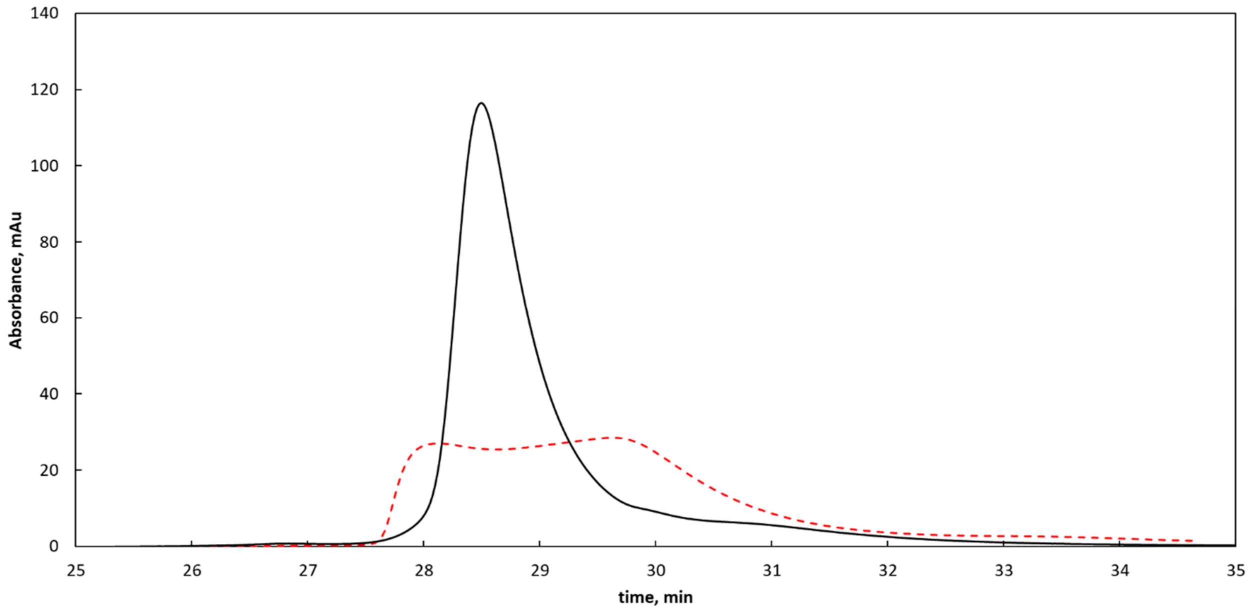

Figure S3. SEC analysis of LYZ solution. Red dashed curve: SEC profile of the LYZ solution after its contact with the hydrogel at 37°C and pH 4.5; black solid curve: SEC profile of the solution of intact LYZ.

Table S5. Comparison of adsorption of mAb2 at different pH and temperatures.

q_P^*, mg g_{gel}^{-1}									
	pH 4.5			pH 7			pH 9.2		
	5°C	20°C	37°C	5°C	20°C	37°C	5°C	20°C	37°C
20BAA-10SMA	0.7	3.2	7.0	8.4	11.9	12.5	3.2	3.7	8.3
10BAA-10SMA	0.3	8.3	8.8	9.8	15.3	17.0	1.3	6.7	9.5
5BAA-10SMA	~0	8.9	9.2	11.4	18.1	26.8	~0	7.2	12.7
20BAA-5SMA	~0	3.3	7.3	5.7	8.7	9.1	0.3	3.8	9.0
10BAA-5SMA	0.2	4.9	7.9	6.1	9.8	10.2	~0	8.0	17.8
5BAA-5SMA	0.3	9.0	9.1	6.6	15.0	23.0	~0	8.7	20.0
20BAA-2SMA	0.4	9.3	7.4	1.6	4.4	8.1	~0	4.0	10.9
10BAA-2SMA	0.7	9.5	7.8	1.9	8.3	9.0	~0	9.7	19.2
5BAA-2SMA	2.7	9.6	8.1	3.1	13.7	21.9	~0	12.2	21.7
